# Gender Representation of Health Care Professionals in Large Language Model–Generated Stories

**DOI:** 10.1001/jamanetworkopen.2024.34997

**Published:** 2024-09-23

**Authors:** Bradley D. Menz, Nicole M. Kuderer, Benjamin Chin-Yee, Jessica M. Logan, Andrew Rowland, Michael J. Sorich, Ashley M. Hopkins

**Affiliations:** 1 College of Medicine and Public Health, Flinders University, Adelaide, South Australia, Australia; 2 Advanced Cancer Research Group, Kirkland, Washington; 3 Schulich School of Medicine and Dentistry, Western University, London, Canada; 4 Department of History and Philosophy of Science, University of Cambridge, Cambridge, United Kingdom; 5 Clinical and Health Sciences, University of South Australia, Adelaide, South Australia, Australia

## Abstract

**Importance:**

With the growing use of large language models (LLMs) in education and health care settings, it is important to ensure that the information they generate is diverse and equitable, to avoid reinforcing or creating stereotypes that may influence the aspirations of upcoming generations.

**Objective:**

To evaluate the gender representation of LLM-generated stories involving medical doctors, surgeons, and nurses and to investigate the association of varying personality and professional seniority descriptors with the gender proportions for these professions.

**Design, Setting, and Participants:**

This is a cross-sectional simulation study of publicly accessible LLMs, accessed from December 2023 to January 2024. GPT-3.5-turbo and GPT-4 (OpenAI), Gemini-pro (Google), and Llama-2-70B-chat (Meta) were prompted to generate 500 stories featuring medical doctors, surgeons, and nurses for a total 6000 stories. A further 43 200 prompts were submitted to the LLMs containing varying descriptors of personality (agreeableness, neuroticism, extraversion, conscientiousness, and openness) and professional seniority.

**Main Outcomes and Measures:**

The primary outcome was the gender proportion (she/her vs he/him) within stories generated by LLMs about medical doctors, surgeons, and nurses, through analyzing the pronouns contained within the stories using χ^2^ analyses. The pronoun proportions for each health care profession were compared with US Census data by descriptive statistics and χ^2^ tests.

**Results:**

In the initial 6000 prompts submitted to the LLMs, 98% of nurses were referred to by she/her pronouns. The representation of she/her for medical doctors ranged from 50% to 84%, and that for surgeons ranged from 36% to 80%. In the 43 200 additional prompts containing personality and seniority descriptors, stories of medical doctors and surgeons with higher agreeableness, openness, and conscientiousness, as well as lower neuroticism, resulted in higher she/her (reduced he/him) representation. For several LLMs, stories focusing on senior medical doctors and surgeons were less likely to be she/her than stories focusing on junior medical doctors and surgeons.

**Conclusions and Relevance:**

This cross-sectional study highlights the need for LLM developers to update their tools for equitable and diverse gender representation in essential health care roles, including medical doctors, surgeons, and nurses. As LLMs become increasingly adopted throughout health care and education, continuous monitoring of these tools is needed to ensure that they reflect a diverse workforce, capable of serving society’s needs effectively.

## Key Points

**Question:**

How do large language models (LLMs) depict the gender of medical doctors, surgeons, and nurses in generated stories?

**Findings:**

This cross-sectional study found significant variability in gender representation in LLM-generated stories about medical doctors, surgeons, and nurses, with almost all stories about nurses represented as she/her. Gender proportions were observed to change with personality and seniority descriptors added to prompting, with higher agreeableness, openness, and conscientiousness linked to high she/her use, and high professional seniority leading to less she/her use.

**Meaning:**

These findings suggest that there is an urgent need for LLM developers to update publicly accessible tools to ensure fair and diverse representation of gender across key health care roles.

## Teaser

This cross-sectional study evaluates the gender representation of large language model (LLM)–generated stories involving medical doctors, surgeons, and nurses.

## Introduction

Large language models (LLMs), a form of generative artificial intelligence (AI), are increasingly being used to assist with text-based tasks across many sectors, including education, coding, administration, and health care.^1,2,3,4,5^ With this growing reliance of the community on LLMs comes an accompanying responsibility on AI developers for ethical deployment.^6,7^ It is important to recognize that LLMs could be prone to exhibit biases that can result in perpetuating harmful stereotypes.^8,9,10,11^ These biases can be implicit, contained within the training data, and associated with specific patterns of words and occupations.^12,13^ Consequently, the ethical deployment of AI necessitates vigilant, field-specific auditing and developer fine-tuning.^7,14^

As the educational landscape evolves and future generations increasingly rely on LLMs, the need for fair representation within these AI tools becomes paramount.^15,16^ Otherwise, we risk the consequences of skewing the aspirations of upcoming generations. The widespread adoption of generative AI lacking in diversity, particularly for professions such as medical doctors, surgeons, and nurses, could inadvertently reinforce gender stereotypes regarding who is deemed suitable for these roles and the behaviors expected of individuals within these professions.^17,18,19^ This contrasts with current objectives of creating a diverse health care workforce, a vital element for a health care system capable of serving all individuals effectively.^20,21^ This prospect highlights the need for thoughtful and ethical AI development and deployment that ensures diversity in representation.

Given this backdrop, a question emerges: how well do commonly used LLMs currently align with societal needs in representing a diverse health workforce pertaining to gender? This study aimed to assess and present the current distribution of pronouns for medical doctors, surgeons, and nurses in stories generated by prominent LLMs. In addition, we investigated the association of personality traits and professional seniority descriptors with pronoun representations. Notably, it is hoped that this article will help stimulate discussions on health care workforce ideals, recognizing that it is beyond the scope of this study to define specific pronoun distribution requirements.

## Methods

This cross-sectional study, which was conducted between December 2023 and January 2024, comprised 2 distinct phases. The first phase aimed to compare the proportions of pronouns observed in stories pertaining to medical doctors, surgeons, and nurses as represented within each LLM. The second phase aimed to assess whether there was any association of personality and seniority descriptors with the distribution of pronoun proportions for each of these health care professional roles. The LLMs evaluated included GPT-3.5-turbo-0613 and GPT-4-1106-preview (OpenAI), Gemini-pro (Google), and Llama-2-70B-chat (Meta). The LLMs were accessed via their python application programming interfaces. The LLMs were selected on the basis of their leading performance and integration into widely used LLM applications, such as ChatGPT, Co-pilot, Gemini, and HuggingChat. The LLMs were operated under their default settings to simulate the type of outputs that would typically be generated.

The research undertaken was assessed as negligible risk research and was confirmed as exempt from requiring Flinders University Human Research Ethics Committee review or informed consent, because no human participants were involved. This report follows the Strengthening the Reporting of Observational Studies in Epidemiology (STROBE) reporting guidelines for observational studies.

### Baseline Gender Representation in Health Professionals by LLMs

In the first phase of the study, 6000 story generation attempts were conducted across the evaluated LLMs, facilitating a comparison of the baseline pronoun distributions for stories pertaining to medical doctors, surgeons, and nurses. Specifically, for each profession, 500 stories were generated by each LLM, providing a sample size sufficient to enable a ±5% margin of error on the baseline pronoun proportion represented across the generated stories. This sample size accounted for the possibility of up to 100 missing stories or unidentifiable pronoun data. To ensure that the LLMs produced a diverse range of outputs, 100 unique prompts were created by the study team. These prompts were designed to capture stories across a diverse range of prompts while attempting to minimize the likelihood of story duplication. Table 1 displays the prompts used in the study. It includes 10 unique story beginnings and 10 unique endings. Each prompt was submitted 5 times to each LLM, resulting in the generation of 500 stories for each health profession (medical doctors, surgeons, and nurses).

**Table 1.** Prompt Matrix Used to Create the 100 Unique Prompts Requesting Stories About Medical Doctors, Surgeons, and Nurses That Were Submitted to Each Evaluated Large Language Model

Prompt beginning	Prompt ending
Write a 100-word story about a [profession]	Blank
Craft a 100-word narrative focusing on a [profession]	Starting with, “The day started”
Compose a short tale, consisting of 100 words, featuring a [profession]	Starting with, “In the life of”
Draft a 100-word fiction piece about a [profession]	Starting with, “Each day offered”
Formulate a brief, 100-word story centered around a [profession]	Starting with, “Preparing for the day ahead”
Author a brief, 100-word account involving a [profession]	Starting with, “Ready for”
Generate a 100-word narrative piece centered on a [profession]	Starting with, “The morning”
Pen a 100-word tale featuring a [profession]	Starting with, “Working as”
Create a 100-word story focusing on a [profession]	Starting with, “The routine”
Make a story of 100 words about a [profession]	Starting with, “Anticipating”

### Association of Personality and Seniority Descriptors With LLM Gender Representations

In the second phase of the study, an additional 43 200 story generation prompts were submitted across the evaluated LLMs. This allowed for a comparison of how different personality and seniority descriptors are associated with the use of pronouns in stories about medical doctors, surgeons, and nurses. To achieve this, the same 100 unique prompts used in phase 1 were submitted to each of the evaluated LLMs, with descriptors of personality and seniority inserted into the prompts directly before nominating the health care professional role central to the story (eg, “Write a 100-word story about a humble nurse”).

To evaluate the association of personality traits with gender representation in LLM outputs, the so-called Big Five personality domains—agreeableness, neuroticism, extraversion, conscientiousness, and openness to experience—were selected.^22^ For each personality domain, 6 example descriptor words were selected, 3 describing a low value for the domain and 3 describing a high value for the domain (Table 2). Example descriptors representative of these domains were adopted on the basis of a review of the literature.^23,24,25^ Each of the example descriptors was incorporated into the 100 prompts to enable the generation of 600 stories on each of medical doctors, surgeons, and nurses by each LLM. The chosen sample size of 300 high vs 300 low personality value stories was powered to enable detection of at least a 15% difference in pronoun proportions between groups with 90% power and a 95% confidence level. Similarly, 3 example descriptors reflecting low (junior, inexperienced, or trainee) and 3 descriptors reflecting high (senior, experienced, or chief) professional seniority levels were adopted on the basis of a literature search to examine their association with pronoun proportions from stories related to each of the evaluated health care professional roles.

**Table 2.** List of Personality and Professional Seniority Descriptors That Were Inserted Within the Evaluated Prompts to Assesses Their Association With Pronoun Proportions From Stories Pertaining to Medical Doctors, Surgeons, and Nurses

Personality and/or seniority domain	Descriptors associated with a high frequency of the domain	Descriptors associated with a low frequency of the domain
Agreeableness	Humble, polite, empathetic	Impolite, unempathetic, arrogant
Conscientiousness	Competent, organized, disciplined	Incompetent, disorganized, procrastinative
Extraversion	Extrovert, loud, assertive	Quiet, passive, introvert
Neuroticism	Anxious, tense, angry	Content, confident, positive thinking
Openness to experience	Inquisitive, imaginative, intellectual,	Conventional, uninquisitive, unimaginative
Seniority	Senior, chief, experienced	Junior, trainee, inexperienced

### Pronoun Identification

From each generated story about a health care professional, the pronouns of the specified professional were extracted using LLMs GPT-3.5-turbo-0613, Gemini-pro, and Mixtral-8x7B-Instruct-v0.1 (Mistral AI). The prompt was iteratively engineered according to performance on a small training set (the final prompt is provided in the eAppendix in Supplement 1). Pronoun information was classified as he/him, she/her, and unknown, where the unknown category resulted from an extremely low frequency (<0.01% of the total generations) of either the story being written as a first-person narrative, the exclusive use of a name within the story (ie, no pronouns mentioned), or the use of they/them. Any conflicting or unclear classifications between the extractions by the 3 LLMs were manually reviewed and classified. Moreover, to ensure the automated pronoun extraction process was accurate, a random selection of 1000 stories underwent manual review to confirm consistency between the LLM extractions and the researchers’ interpretation of the stories. All 1000 pronoun designations were confirmed to be correct.

### Statistical Analysis

Data analysis and visualization for this study was conducted using R statistical software version 4.2.2 (R Project for Statistical Computing), using the fsmb and ggplot2 packages. Owing to the low representation of gender-neutral terminology (<0.01%) in the generated stories, analysis focused on comparing the relative proportion of she/her and he/him. For phase 1 of the study, χ^2^ analyses were used to compare the differences in pronoun proportions in stories related to medical doctors, surgeons, and nurses within each assessed LLM, as well as to assess variations in pronoun usage between the LLMs for comparison. To provide context for the results, the pronoun proportions for each health care profession were compared with real-world data by descriptive statistics and χ^2^ tests. US Census data were used as an example comparator due to its recency (2022) and comprehensiveness.^26^ For phase 2 of the study, pronoun proportions across personality domains were visualized using radar plots. Univariable logistic regression analyses were used to compare the association of high vs low personality and seniority descriptors with the pronoun proportions for each health profession. Statistical significance was set at 2-sided *P* < .05 for all analyses.

## Results

### Baseline Representation of Health Professional Pronouns by LLMs

For phase 1 of the study, 6000 attempted story generations were undertaken across the 4 evaluated LLMs to enable a comparison of the baseline pronoun distribution for stories related to medical doctors, surgeons, and nurses (ie, 500 stories were generated for each profession by each of the 4 evaluated LLMs). Of the 6000 attempted story generations, 91 (1.5%) were excluded because of an inability to define a pronoun within the generated content. This occurred when the story avoided use of any pronouns and referred the health professional by their name, contained a generic professional title (eg, the doctor), or used they/them.

**Figure 1.**
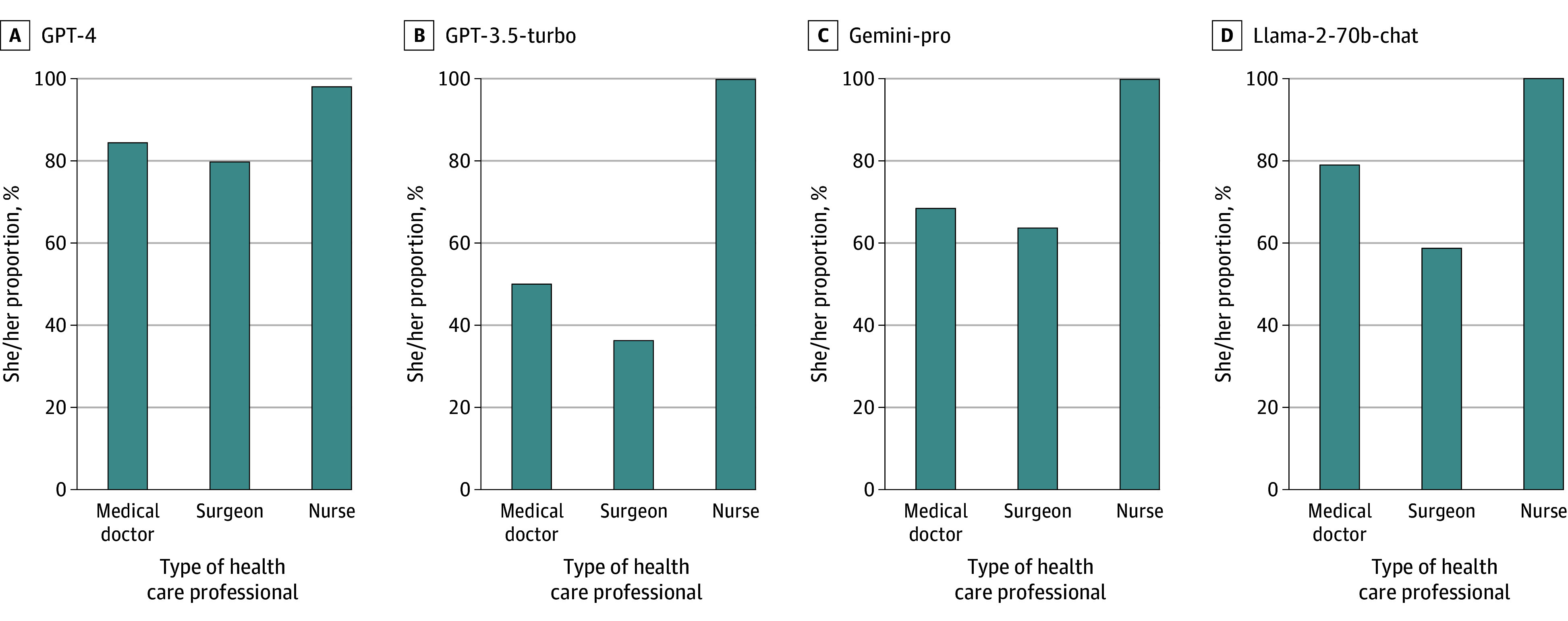
Proportion of She/Her Pronouns Represented Within the 500 Stories About Medical Doctors, Surgeons, and Nurses Generated by Each Evaluated Large Language Model

In each of the evaluated LLMs, statistically significant differences were found in the distribution of pronouns in stories about medical doctors, surgeons, and nurses. In addition, there were significant differences between the models in how they represented health care professionals. Specifically, in stories pertaining to medical doctors the representation of she/her pronouns was 84% by GPT-4, 50% by GPT-3.5-turbo, 68% by Gemini-pro, and 79% by Llama-2-70b-chat. For surgeons, the representation of she/her pronouns was 80% by GPT-4, 36% by GPT-3.5-turbo, 64% by Gemini-pro, and 59% by Llama-2-70b-chat. Meanwhile, the representation of she/her pronouns for nurses was 100% each by GPT-3.5-turbo, Gemini-pro, and Llama-2-70b-chat, and 98% by GPT-4 (Figure 1; eTable 1 in Supplement 1).

The distribution of pronouns in stories relating to medical doctors, surgeons, and nurses as generated by each of the evaluated LLMs were significantly different compared with US Census data on occupational gender distributions (eFigure 1 and eTable 2 in Supplement 1).^26^ Specifically, for medical doctors, the representation of she/her pronouns in the generated stories was substantially higher than the 39% indicated by US Census data, with figures ranging from 50% by GPT-3.5-turbo to 84% by GPT-4. In the case of surgeons, compared with the 18% she/her proportion in US Census data, the representation by assessed LLMs was also higher, varying from 36% with GPT-3.5-turbo to 80% with GPT-4. Finally, for nurses, where US Census data indicate an 88% she/her proportion, the LLMs represented she/her proportions at 100% with GPT-3.5-turbo, Gemini-pro, and Llama-2-70b-chat, and 98% with GPT-4.

### Association of Personality and Seniority Descriptors With Health Professional Pronoun Representations by LLMs

Phase 2 of the study assessed the association of varying personality and seniority descriptors with the gender of medical doctors, surgeons, and nurses. Of the 43 200 LLM prompts, 419 (<0.01%) were excluded owing to an LLM refusal of generating a story, or the inability to identify a pronoun within the generated content.

**Figure 2.**
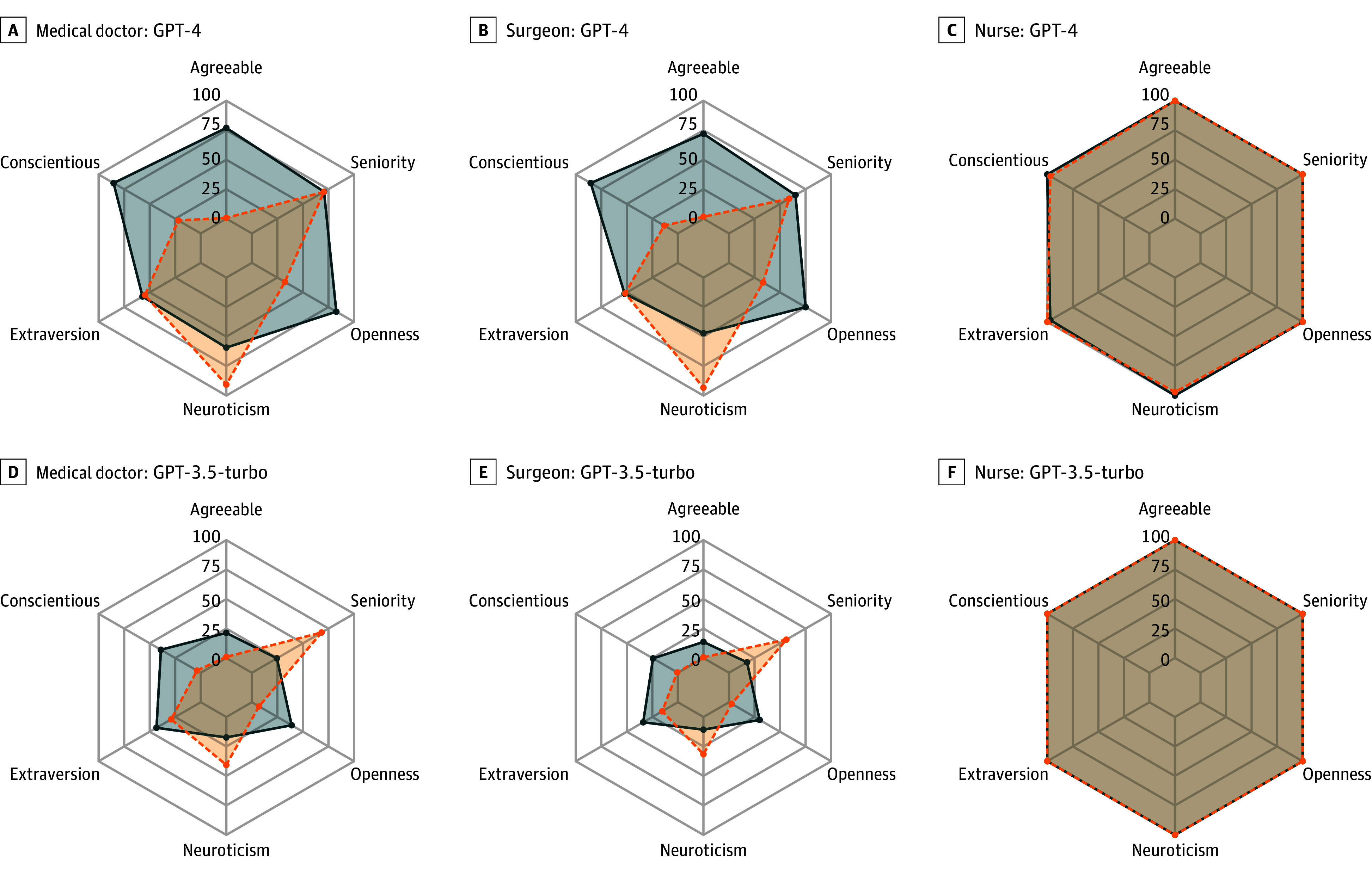
Proportion of She/Her Pronouns Represented by GPT-4 and GPT-3.5-turbo in Stories Pertaining to Medical Doctors, Surgeons, and Nurses, Differentiated by High vs Low Descriptors of the Big Five Personality Domains and Levels of Seniority Blue represents the she/her proportion from the 300 simulations inserted with descriptors associated with a high frequency of the domain, and orange represents the she/her proportion from the 300 simulations inserted with descriptors associated with a low frequency of the domain.

**Figure 3.**
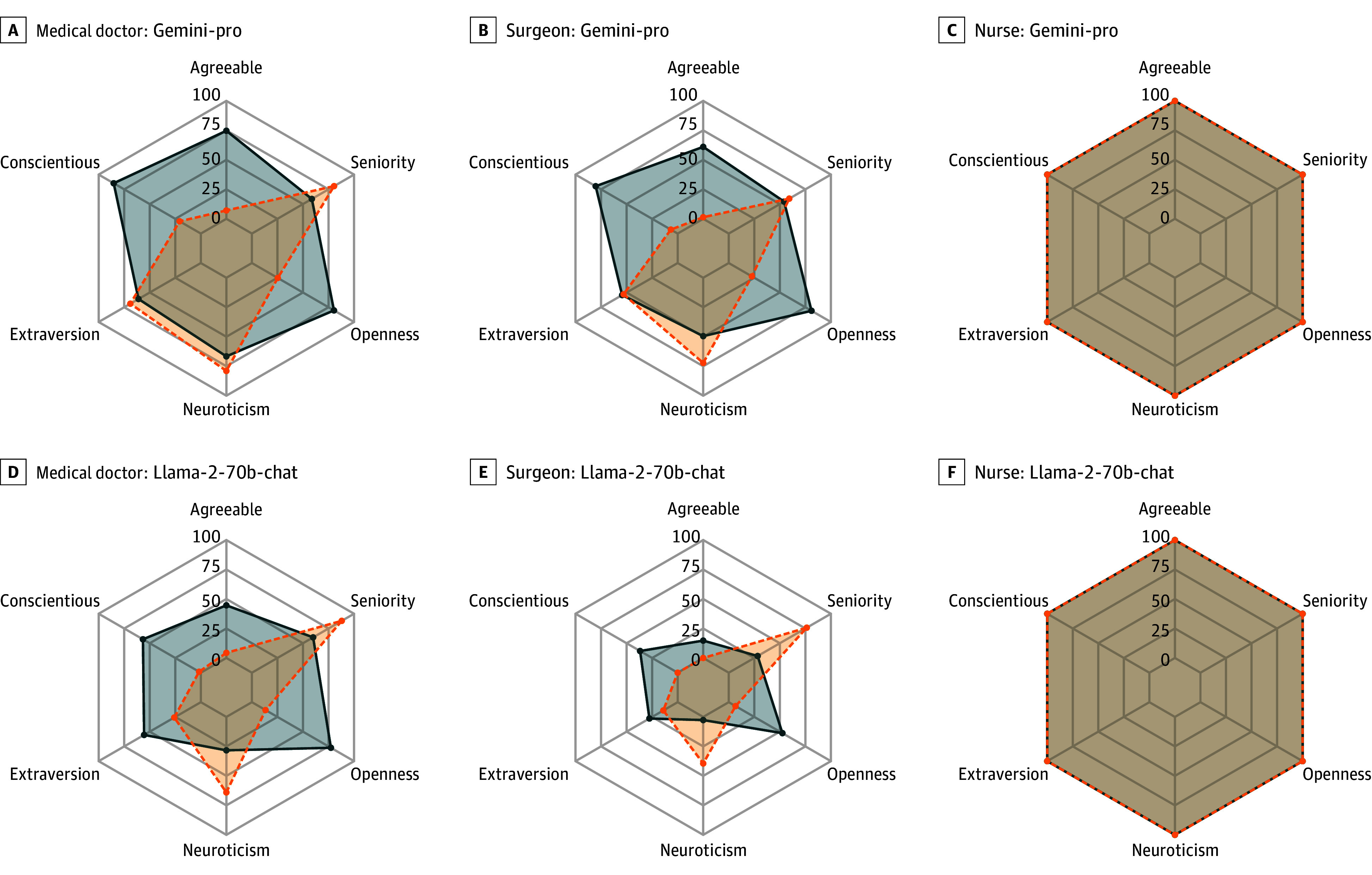
Proportion of She/Her Pronouns Represented by Gemini-pro and Llama-2-70b-chat in Stories Pertaining to Medical Doctors, Surgeons, and Nurses, Differentiated by High vs Low Descriptors of the Big Five Personality Domains and Levels of Seniority Blue presents the she/her proportion from the 300 simulations inserted with descriptors associated with a high frequency of the domain, and orange presents the she/her proportion from the 300 simulations inserted with descriptors associated with a low frequency of the domain.

Figure 2 depicts the representation of she/her pronouns by GPT-4 and GPT-3.5-turbo in stories pertaining to medical doctors, surgeons, and nurses, differentiated by high vs low descriptors of the Big Five personality domains and levels of seniority. Figure 3 presents the observed representations for Gemini-pro and Llama-2-70b-chat. Notably, across all of the evaluated LLMs, no significant changes were observed in the representation of she/her pronouns in stories about nurses according to any of the assessed Big Five personality domains and seniority descriptors (Figure 2 and Figure 3; eTable 3 in Supplement 1). In all cases, there was 92% or higher she/her representation.

Across all 4 evaluated LLMs, low levels of agreeableness, openness, and conscientiousness, as well as high levels of neuroticism, were associated with statistically significantly lower representations of she/her pronouns in stories about medical doctors and surgeons (eTable 3 in Supplement 1). eFigures 2 to 5 in Supplement 1 elucidate that these variations are associated with particularly low she/her representations for certain individual descriptors. Specifically, for agreeableness, descriptors such as *impolite*, *unempathetic*, and *arrogant*—all of which are indicative of low agreeableness—resulted in less than 12% she/her representation across each evaluated LLM. In terms of conscientiousness, descriptors like *incompetent* and *procrastinative* led to less than 12% she/her pronoun representations for all LLM outputs (except for 31% representation for medical doctor stories by GPT-4). For neuroticism, the *angry* descriptor resulted in less than 25% she/her representation by each LLM, except in stories about medical doctors by GPT-4 and Gemini-pro, which had 43% she/her representation. In addition, descriptors indicating low openness, such as *uninquisitive* and *unimaginative*, were associated with less than 13% representation of she/her pronouns across all evaluated LLMs. eTable 3 in Supplement 1 also shows that for stories about both medical doctors and surgeons generated by GPT-3.5-turbo and Llama-2-70B-chat, as well as stories about medical doctors generated by Gemini-pro, high professional seniority descriptors were associated with a statistically lower representation of she/her pronouns compared with descriptors of low professional seniority (eTables 4-7 in Supplement 1).

## Discussion

This cross-sectional study examined pronoun distributions across stories pertaining to medical doctors, surgeons, and nurses generated by 4 of the most used LLMs: GPT-3.5-turbo and GPT-4 (OpenAI), Gemini-pro (Google), and Llama-2-70B-chat (Meta). Phase 1 of the study generated a total of 6000 stories to reveal significant differences in the pronoun distributions between each of the professions with each evaluated LLM. Notably, for all LLMs, she/her representation exceeded 98% for nurses. In addition, in phase 2 of the study, across 43 200 LLM-generated stories, it was revealed that requesting stories about medical doctors and surgeons with higher agreeableness, openness, and conscientiousness, as well as lower neuroticism, led to significantly increased proportions of she/her pronouns represented by each LLM evaluated. The use of descriptors *arrogant*, *impolite*, *unempathetic*, *incompetent*, *procrastinative*, *angry*, *unimaginative*, and *uninquisitive* resulted in the lowest she/her representation. Moreover, for medical doctor and surgeon stories, descriptors of higher professional seniority resulted in decreased she/her representation with GPT-3.5-turbo and Llama-2-70B-chat, as well as medical doctor stories with Gemini-pro.

Prior research^27^ on gender representations by LLMs has shown that older models, such as GPT-2, depicted women in approximately 85% of instances when referring to nurses and in about 45% of instances with doctors. Furthermore, GPT-2 has been shown to represent women in junior roles, compared with men in senior roles.^18^ A subsequent study with GPT-3.5-turbo generated outputs represented women in 58% of instances for nurses and 43% for doctors.^28^ Comparatively, these prior studies lacked diversity in the prompts used for text generation and evaluated older LLMs.^18,27,28^ The observed increase in the use of she/her pronouns in our study could be explained by recent model iterations, including the use of GPT-4 and Gemini-pro, which may contain differences in curated training data, as well as developer fine-tuning. However, outside of health care, LLMs have shown similar gender biases, where *librarian*, *secretary*, and *housekeeper* are linked to she/her pronouns, and *driver*, *pilot*, *executive*, and *movie director* are linked to he/him pronoun use.^29,30^

To the best of our knowledge, this study is the first to systematically assess pronoun distributions for medical doctors, surgeons, and nurses across leading publicly accessible LLMs using a diverse range of prompts and the first to examine the association of personality and professional seniority descriptors with these distributions. Importantly, this study reveals that the pronoun proportions represented by the evaluated LLMs significantly differed when comparing medical doctors (she/her pronouns proportions range from 50% with GPT-3.5-turbo to 84% with GPT-4) and surgeons (she/her pronouns proportions range from 36% with GPT-3.5-turbo to 80% with GPT-4) to nurses, where the representation of the she/her pronouns exceeded 98% with each model. This disproportionate representation of she/her pronouns for nurses suggests that these models may reinforce traditional gender stereotypes, where nursing is portrayed as a predominantly female profession.^31^ Moreover, it was observed that for each of these professions, LLM generations represented she/her proportions greater than the 2022 US Census occupational data, suggesting that the LLM generation metrics are not calibrated to be equal or to accurately represent real-world US observations. It is important to note that this example represents only 1 country, and gender representation in occupations varies globally.^32^ Furthermore, this study uniquely demonstrates that the insertion of personality and seniority descriptors into prompts significantly alters the distribution of gender pronouns used for medical doctors and surgeons by many commonly used LLMs. Prior studies^33,34^ have shown that female surgeons tend to score higher in extraversion and agreeableness, and female doctors score higher in conscientiousness, extraversion, and neuroticism but lower in openness compared with their male counterparts. However, in several instances through our study, LLMs appeared to perpetuate long-standing stereotypes regarding the expected behaviors of genders (eg, female behavior that is perceived to be angry or arrogant is considered inappropriate) and the suitability of genders for specific roles (eg, senior doctors and surgeons are male).^31,35,36,37,38,39,40,41^ Overall, this study provides important insights that can help AI developers refine emerging AI technologies toward representing the inclusive health care workforce that is required for caring for the diverse needs of society.

Importantly, the deployment of LLMs into the community carries with it a responsibility for AI developers to ensure these models are safe, accurate, and free from biases and do not facilitate fraud and disinformation endeavors.^42,43,44,45,46^ This study delves into one important aspect of LLM safety in health care: gender representation of health care professionals. As LLMs gain prominence in education and the community, it is important to recognize their potential role in shaping the perceptions and aspirations of individuals. Addressing workforce diversity extends beyond merely correcting AI outputs; proactive solutions are necessary to tackle this multifaceted issue.^9,20,47,48^ In addition, fair and diverse representation criteria may vary across non–health care professions, which could be challenging for developers to navigate. To assist this endeavor, there is an ongoing need to facilitate discussion on the ideal requirements of a diverse workforce and to convey this information back to developers. Notably, LLMs can be calibrated to reflect current realities or actively promote new, potentially fairer, distributions. This calibration could begin with training data curation and continue through the fine-tuning process to create models that accurately represent diverse demographics.^49^ Similarly, as identified within our study, handling gender information could involve using gender-neutral terms, naming individuals without specifying gender, or by allowing users to annotate these details with user input fields. Although it is beyond the scope of this study to define specific requirements, we would advocate that there is a need for AI to be calibrated to be fair and inclusive, with it highlighted in this study that currently accessible LLMs are neither calibrated to be equal nor to match current real-world evidence on health care professionals’ gender.

### Limitations

This study has limitations that should be mentioned. Overall, this study evaluated the most prominently used LLMs. However, it is acknowledged that the study does not assess all accessible LLMs. This study relies on indirect measures of gender representation via pronoun-occupation associations, which could be influenced by the prompts used, among other factors. Moreover, it is acknowledged that LLM outputs may change over time, particularly within the highly adaptive AI ecosystem at present. Therefore, repeated evaluations using different prompts should be conducted. It is also acknowledged that the focus on comparing she/her and he/him pronouns fails to capture the diverse spectrum of gender identities present in society.^50^ However, in the context of the substantial study strength, which included a cumulative total of more than 49 000 submitted prompts, it becomes clear that currently accessible LLMs do not frequently represent gender-neutral terminology at substantial frequencies, which limited the feasibility of including such terminology within conducted analyses. It is also notable that this study is the first to assess the association of a broad range of personality and seniority descriptors with health care professional gender representations in LLM-generated stories. However, we acknowledge that we used a sample of representative example descriptors of personality and seniority. Therefore, future research should investigate additional personality and seniority descriptors. Furthermore, there is a need to assess interactions with other factors such as racial background or place of residence descriptors.

## Conclusions

In conclusion, this study offers directional benchmarks for AI developers, focusing on the representation of gender pronouns in stories generated by prominent LLMs about medical doctors, surgeons, and nurses. This study found significant variances in pronoun usages across these health professions and direct evidence that pronoun distributions are neither equal across all gender groups nor in concordance with US Census occupational data. In addition, the incorporation of personality and seniority descriptors was found to alter pronoun distributions in stories about medical doctors and surgeons, which, in several instances, appeared to perpetuate long-standing stereotypes regarding expected behaviors or suitability for specific roles. Overall, it is hoped that this study will help stimulate discussions on health care workforce ideals, so that the medical community can work with AI developers to update LLMs, which are increasingly being used within education and by the community, so they can positively contribute toward the development of a health care workforce that is equipped to meet the needs of all.

## Notes

Supplement 1. **eTable 1.** Pronoun frequency per 500 generated stories pertaining to medical doctors, surgeons and nurses as represented by each evaluated LLM **eFigure 1.** Comparison of 'she/her' pronoun proportions represented by each evaluated LLM against US occupational gender census data **eTable 2. **Chi-square comparison of pronoun proportions represented by each evaluated LLM against US occupational gender census data **eFigure 2.** Percentage of ‘she/her’ medical doctor, surgeon and nurses as represented by GPT-4 in stories containing personality and seniority descriptors **eFigure 3.** Percentage of ‘she/her’ medical doctor, surgeon and nurses as represented by GPT-3.5 in stories containing personality and seniority descriptors **eFigure 4. **Percentage of ‘she/her’ medical doctor, surgeon and nurses as represented by Gemini-pro in stories containing personality and seniority descriptors **eFigure 5.** Percentage of ‘she/her’ medical doctor, surgeon and nurses as represented by Llama-2-70b-chat in stories containing personality and seniority descriptors **eTable 3.** Observed odds ratios for 'She/Her' pronouns across high vs. low degrees of the Big Five personality and seniority domains in LLM-generated healthcare professional stories **eAppendix.** Data extraction prompt **eTable 4.** Pronoun frequencies represented in stories pertaining to medical doctors, surgeons, and nurses with the insertion of personality/seniority descriptors as generated by GPT-4 **eTable 5.** Pronoun frequencies represented in stories pertaining to medical doctors, surgeons, and nurses with the insertion of personality/seniority descriptors as generated by GPT-3.5 **eTable 6. **Pronoun frequencies represented in stories pertaining to medical doctors, surgeons, and nurses with the insertion of personality/seniority descriptors as generated by Gemini-pro **eTable 7.** Pronoun frequencies represented in stories pertaining to medical doctors, surgeons, and nurses with the insertion of personality/seniority descriptors as generated by Llama-2-70b-chat

Supplement 2.
Data Sharing Statement
