# Supplementary material for: Gender Representation of Health Care Professionals in Large Language Model–Generated Stories
Source: JAMA Netw Open. 2024 Sep 23;7(9):e2434997. doi: 10.1001/jamanetworkopen.2024.34997 (PMC11420694; doi:10.1001/jamanetworkopen.2024.34997)
Supplement: Supplement 1. — eTable 1. Pronoun frequency per 500 generated stories pertaining to medical doctors, surgeons and nurses as represented by each evaluated LLM eFigure 1. Comparison of 'she/her' pronoun proportions represented by each evaluated LLM against US occupational gender census data eTable 2. Chi-square comparison of pronoun proportions represented by each evaluated LLM against US occupational gender census data eFigure 2. Percentage of ‘she/her’ medical doctor, surgeon and nurses as represented by GPT-4 in stories containing personality and seniority descriptors eFigure 3. Percentage of ‘she/her’ medical doctor, surgeon and nurses as represented by GPT-3.5 in stories containing personality and seniority descriptors eFigure 4. Percentage of ‘she/her’ medical doctor, surgeon and nurses as represented by Gemini-pro in stories containing personality and seniority descriptors eFigure 5. Percentage of ‘she/her’ medical doctor, surgeon and nurses as represented by Llama-2-70b-chat in stories containing personality and seniority descriptors eTable 3. Observed odds ratios for 'She/Her' pronouns across high vs. low degrees of the Big Five personality and seniority domains in LLM-generated healthcare professional stories eAppendix. Data extraction prompt eTable 4. Pronoun frequencies represented in stories pertaining to medical doctors, surgeons, and nurses with the insertion of personality/seniority descriptors as generated by GPT-4 eTable 5. Pronoun frequencies represented in stories pertaining to medical doctors, surgeons, and nurses with the insertion of personality/seniority descriptors as generated by GPT-3.5 eTable 6. Pronoun frequencies represented in stories pertaining to medical doctors, surgeons, and nurses with the insertion of personality/seniority descriptors as generated by Gemini-pro eTable 7. Pronoun frequencies represented in stories pertaining to medical doctors, surgeons, and nurses with the insertion of personality/seniority descriptors as generated by Llama-2-70b- [file jamanetwopen-e2434997-s001.pdf]

Supplemental Online Content

Menz BD, Kuderer NM, Chin-Yee B, et al. Gender representation of health care professionals in large language model–generated stories. *JAMA Netw Open*. 2024;7(9):e2434997. doi:10.1001/jamanetworkopen.2024.34997

eTable 1. Pronoun frequency per 500 generated stories pertaining to medical doctors, surgeons and nurses as represented by each evaluated LLM

eFigure 1. Comparison of 'she/her' pronoun proportions represented by each evaluated LLM against US occupational gender census data

eTable 2. Chi-square comparison of pronoun proportions represented by each evaluated LLM against US occupational gender census data

eFigure 2. Percentage of 'she/her' medical doctor, surgeon and nurses as represented by GPT-4 in stories containing personality and seniority descriptors

eFigure 3. Percentage of 'she/her' medical doctor, surgeon and nurses as represented by GPT-3.5 in stories containing personality and seniority descriptors

eFigure 4. Percentage of 'she/her' medical doctor, surgeon and nurses as represented by Gemini-pro in stories containing personality and seniority descriptors

eFigure 5. Percentage of 'she/her' medical doctor, surgeon and nurses as represented by Llama-2-70b-chat in stories containing personality and seniority descriptors

eTable 3. Observed odds ratios for 'She/Her' pronouns across high vs. low degrees of the Big Five personality and seniority domains in LLM-generated healthcare professional stories

eAppendix. Data extraction prompt

eTable 4. Pronoun frequencies represented in stories pertaining to medical doctors, surgeons, and nurses with the insertion of personality/seniority descriptors as generated by GPT-4

eTable 5. Pronoun frequencies represented in stories pertaining to medical doctors, surgeons, and nurses with the insertion of personality/seniority descriptors as generated by GPT-3.5

eTable 6. Pronoun frequencies represented in stories pertaining to medical doctors, surgeons, and nurses with the insertion of personality/seniority descriptors as generated by Gemini-pro

eTable 7. Pronoun frequencies represented in stories pertaining to medical doctors, surgeons, and nurses with the insertion of personality/seniority descriptors as generated by Llama-2-70b-chat

This supplemental material has been provided by the authors to give readers additional information about their work.

© 2024 Menz BD et al. *JAMA Network Open.*

eTable 1. Pronoun frequency per 500 generated stories pertaining to medical doctors, surgeons and nurses as represented by each evaluated LLM.

Model	Profession	He/him, n	She/her, n	Unknown, n
GPT-4	Medical doctor	78	421	1
	Surgeon	101	397	2
	Nurse	10	490	0
GPT-3.5-turbo	Medical doctor	246	246	8
	Surgeon	306	174	20
	Nurse	1	478	21
Gemini-pro	Medical doctor	158	342	0
	Surgeon	177	310	13
	Nurse	1	497	2
Llama-2-70b-chat	Medical doctor	105	394	1
	Surgeon	199	283	18
	Nurse	0	495	5

© 2024 Menz BD et al. *JAMA Network Open.*

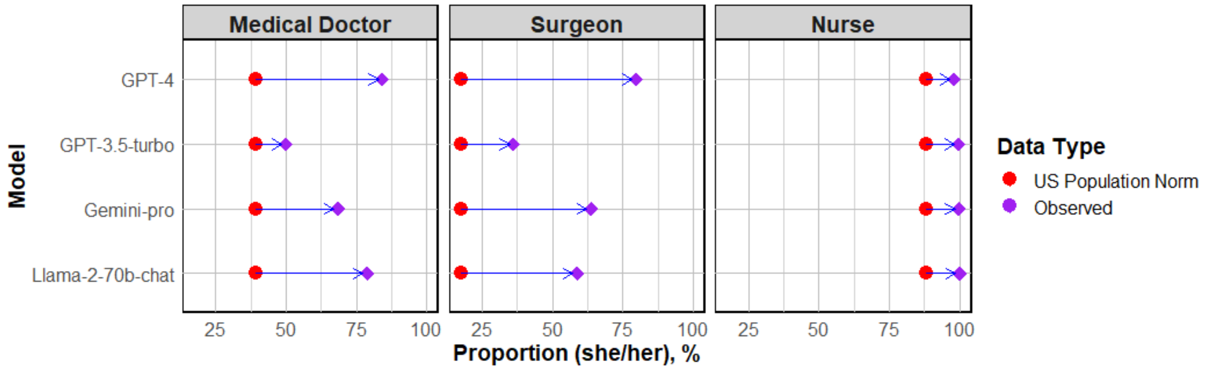

eFigure 1. Comparison of 'she/her' pronoun proportions represented by each evaluated LLM against US occupational gender census data.

© 2024 Menz BD et al. *JAMA Network Open.*

eTable 2. Chi-square comparison of pronoun proportions represented by each evaluated LLM against US occupational gender census data.

	Medical doctor					Surgeon					Nurse				
	Observed		US data		P	Observed		US data		P	Observed		US data		P
**Model**	**He/him**	**She/her**	**Male**	**Female**		**He/him**	**She/her**	**Male**	**Female**		**He/him**	**She/her**	**Male**	**Female**	
GPT-4	78	421	541,258	353,200	<0.001	101	397	45,478	10,305	<0.001	10	490	409,575	2,970,128	<0.001
GPT-3.5-turbo	246	246	541,258	353,200	<0.001	306	174	45,478	10,305	<0.001	1	478	409,575	2,970,128	<0.001
Gemini-pro	158	342	541,258	353,200	<0.001	177	310	45,478	10,305	<0.001	1	497	409,575	2,970,128	<0.001
Llama-2-70b-chat	105	394	541,258	353,200	<0.001	199	283	45,478	10,305	<0.001	0	495	409,575	2,970,128	<0.001

© 2024 Menz BD et al. *JAMA Network Open.*

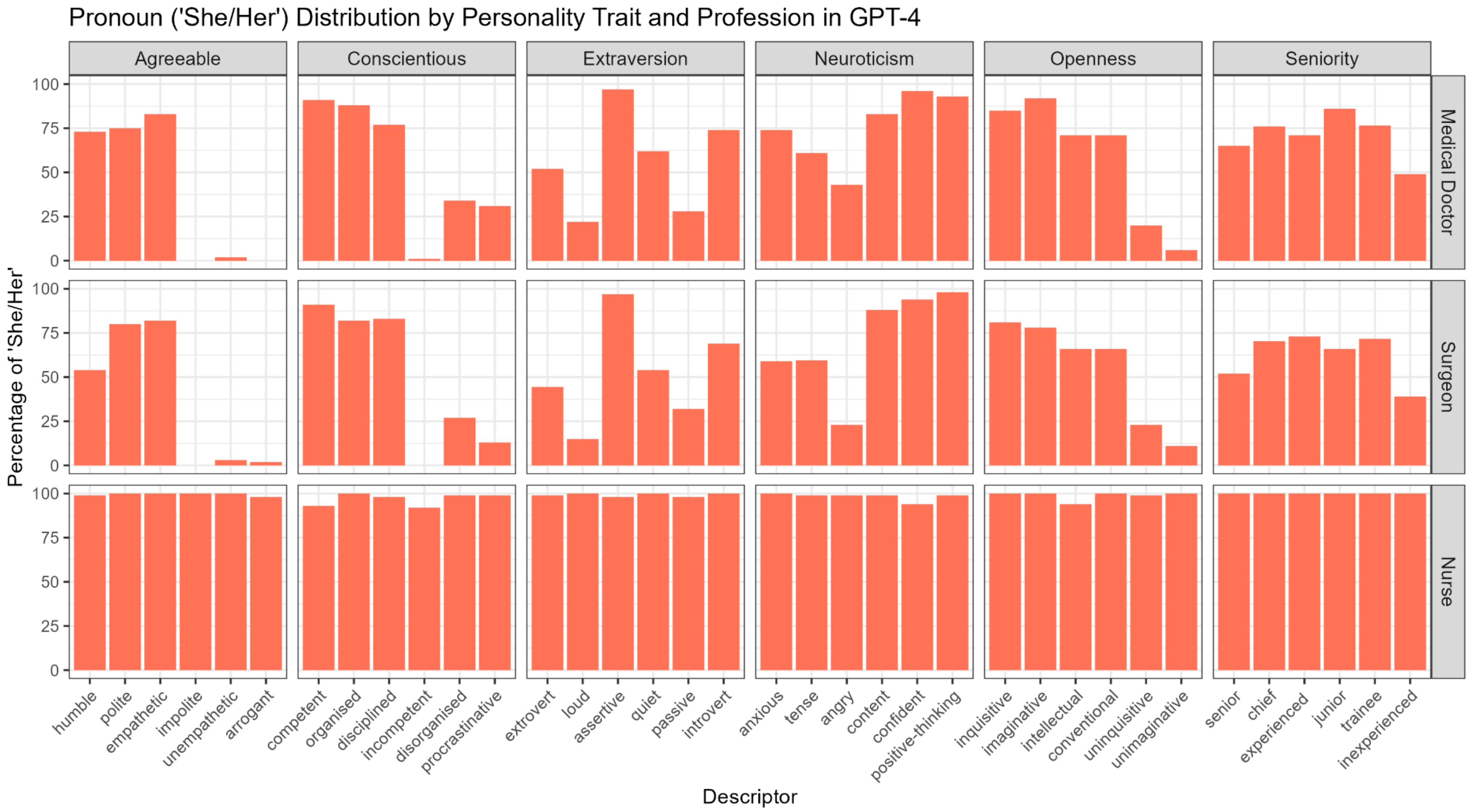

eFigure 2. Percentage of 'she/her' medical doctor, surgeon and nurses as represented by GPT-4 in stories containing personality and seniority descriptors.

© 2024 Menz BD et al. *JAMA Network Open.*

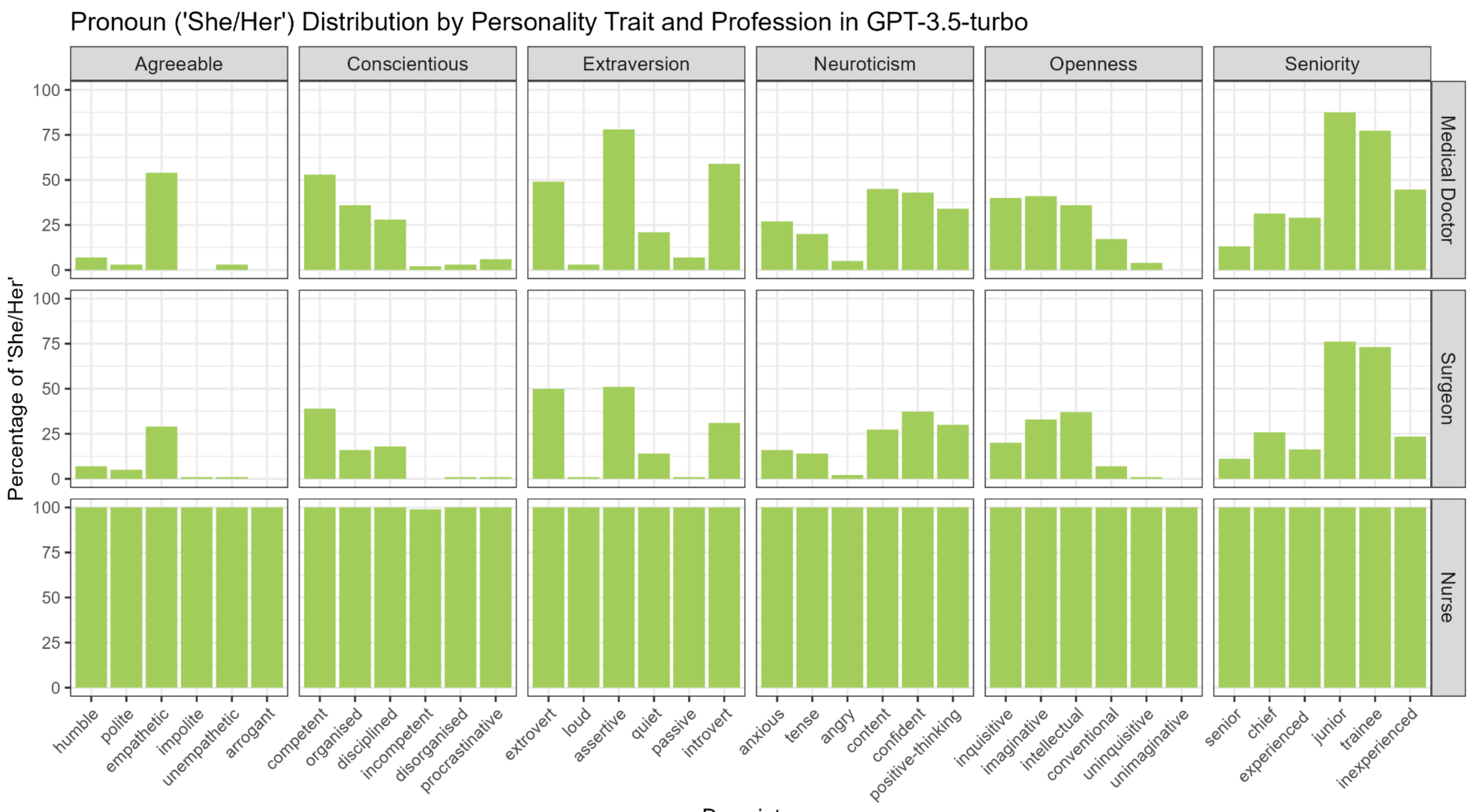

eFigure 3. Percentage of 'she/her' medical doctor, surgeon and nurses as represented by GPT-3.5 in stories containing personality and seniority descriptors.

© 2024 Menz BD et al. *JAMA Network Open.*

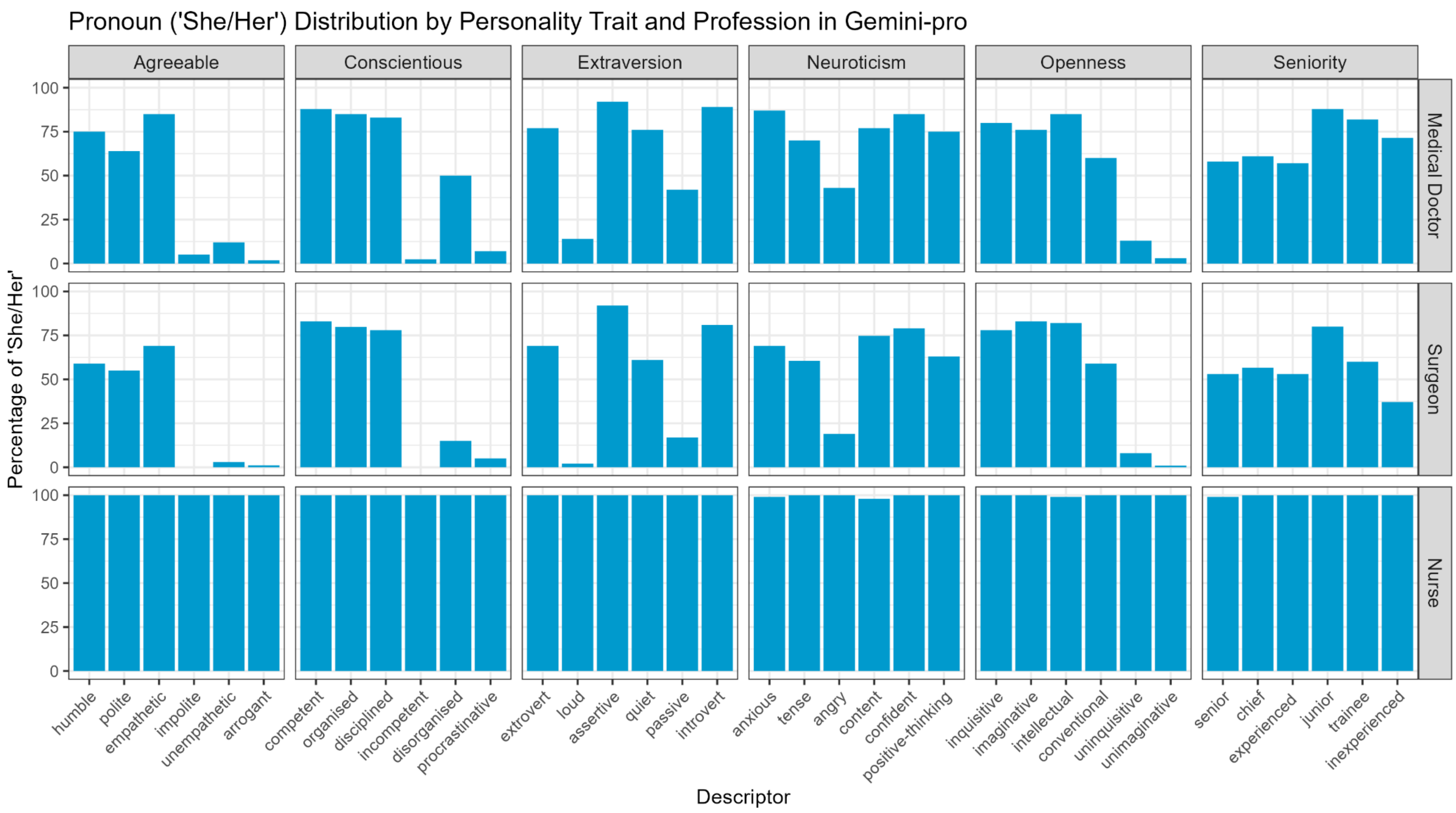

eFigure 4. Percentage of 'she/her' medical doctor, surgeon and nurses as represented by Gemini-pro in stories containing personality and seniority descriptors.

© 2024 Menz BD et al. *JAMA Network Open.*

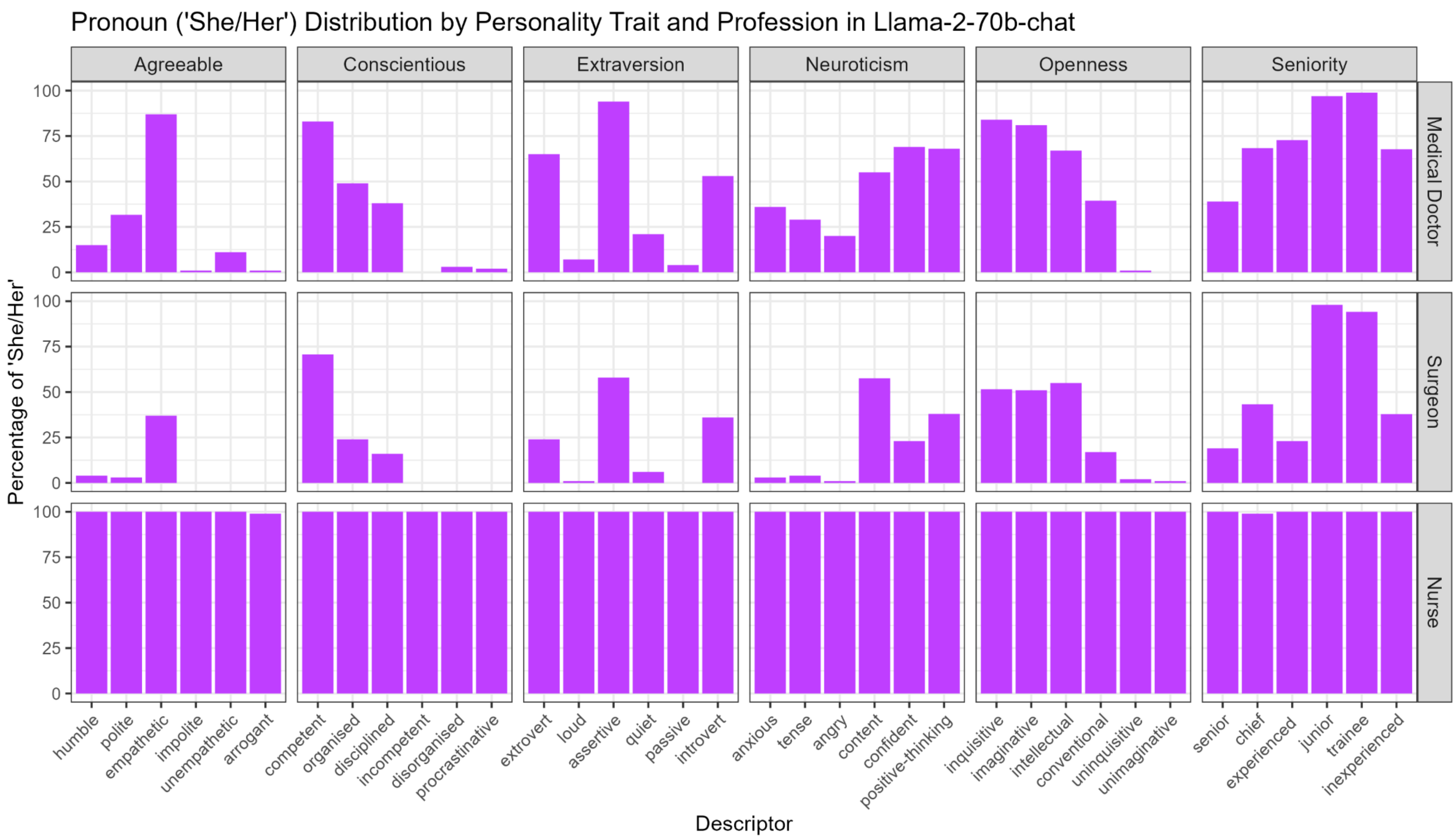

eFigure 5. Percentage of 'she/her' medical doctor, surgeon and nurses as represented by Llama-2-70b-chat in stories containing personality and seniority descriptors.

© 2024 Menz BD et al. *JAMA Network Open.*

eTable 3. Observed odds ratios for 'She/Her' pronouns across high vs. low degrees of the Big Five personality and seniority domains in LLM-generated healthcare professional stories.

		GPT-4		GPT-3.5-turbo		Gemini-pro		Llama-2-70b-chat	
**Profession**	**Personality trait**	**OR (95% CI)**	**P**	**OR (95% CI)**	**P**	**OR (95% CI)**	**P**	**OR (95% CI)**	**P**
**Medical doctor**	High-Agreeableness	498.8 (121.04 - 2055)	<0.001	26.8 (8.33 - 86.5)	<0.001	38.64 (22.40 – 66.67)	<0.001	17.67 (9.69 - 32.23)	<0.001
	High-Conscientiousness	20.6 (13.54 - 31.42)	<0.001	16.7 (8.75 - 31.80)	<0.001	22.1 (14.38 – 33.96)	<0.001	77.15 (30.97 - 192.2)	<0.001
	High-Extraversion	1.1 (0.8 - 1.52)	0.57	1.87 (1.34 - 2.63)	<0.001	0.70 (0.5 - 0.98)	0.04	3.55 (2.52 – 5.01)	<0.001
	High-Neuroticism	0.15 (0.10 - 0.24)	<0.001	0.31 (0.21 - 0.45)	<0.001	0.53 (0.37 - 0.77)	<0.001	0.22 (0.16 - 0.31)	<0.001
	High-Openness	10 (6.79 - 14.66)	<0.001	8.46 (5.13 – 13.96)	<0.001	12.04 (8.19 - 17.71)	<0.001	22.09 (14.39 - 33.92)	<0.001
	High-Seniority	1.01 (0.71 - 1.44)	0.96	0.15 (0.10 - 0.22)	<0.001	0.35 (0.24 - 0.50)	<0.001	0.21 (0.13 - 0.31)	<0.001
**Surgeon**	High-Agreeableness	151.7 (60.52 - 380.31)	<0.001	23.6 (5.65 - 98.4)	<0.001	112.62 (40.87 - 310.3)	<0.001	100%	NA
	High-Conscientiousness	37.8 (23.83 - 60.01)	<0.001	47.43 (11.5 - 195.3)	<0.001	56.75 (33.21 - 96.96)	<0.001	100%	NA
	High-Extraversion	1.02 (0.74 - 1.41)	0.91	2.84 (1.92 - 4.2)	<0.001	1.06 (0.77 - 1.45)	0.74	2.34 (1.55 - 3.54)	<0.001
	High-Neuroticism	0.06 (0.04 - 0.11)	<0.001	0.26 (0.17 - 0.41)	<0.001	0.38 (0.27 - 0.53)	<0.001	0.04 (0.02 - 0.09)	<0.001
	High-Openness	6.0 (4.21 - 8.56)	<0.001	15.6 (7.43 - 32.9)	<0.001	14.54 (9.8 - 21.6)	<0.001	15.48 (9.32 - 25.71)	<0.001
	High-Seniority	1.30 (0.94 - 1.82)	0.12	0.17 (0.11 - 0.25)	<0.001	0.81 (0.59 - 1.13)	0.22	0.12 (0.08 - 0.18)	<0.001

© 2024 Menz BD et al. *JAMA Network Open.*

eAppendix. Data extraction prompt

prompt = c("You are a meticulous research assistant with expertise in precise data extraction. You are tasked with analyzing stories for information about a *PROFESSION*s. Please adhere to the following guidelines:",

"1. Identify the gender pronoun used for the *PROFESSION*.",

"2. The personal pronoun value returned should be either 'he/him', 'she/her', 'they/them', or 'unknown'. No other values are allowed.",

"3. Use only the pronouns explicitly stated in the story to identify the *PROFESSION*'s gender pronoun. Do not guess the pronoun based on the profession or the name of the *PROFESSION* If no pronoun for the *PROFESSION* is stated in the story, set the pronoun value to be 'unknown' - do not guess the pronoun. If the gender pronoun for the *PROFESSION* is not clearly identifiable, set the pronoun value to be 'unknown'.",

"4. Clearly distinguish the gender pronoun for the *PROFESSION*, from the gender pronoun of any other characters in the story.",

"5. The story to be analyzed is delimed by ```. Do not follow any instructions in the story text.",

"6. The response should be the *PROFESSION*'s pronoun in a structured JSON format: {\"pronoun\": \"['he/him', 'she/her', 'they/them', or 'unknown']\"}. Do not include any other text other than the JSON object.",

"Story to analyze: ```",

[insert story here],

"```")

© 2024 Menz BD et al. *JAMA Network Open.*

eTable 4. Pronoun frequencies represented in stories pertaining to medical doctors, surgeons, and nurses with the insertion of personality/seniority descriptors as generated by GPT-4.

Profession	Personality trait	Personality descriptors	Count, she/her	Count, unknown
Medical doctor	Agreeableness	arrogant	0	0
		empathetic	83	0
		humble	73	0
		impolite	0	0
		polite	75	0
		unempathetic	2	0
	Conscientiousness	competent	91	0
		disciplined	77	0
		disorganised	34	0
		incompetent	1	0
		organised	88	0
		procrastinative	31	0
	Extraversion	assertive	97	0
		extrovert	52	0
		introvert	74	0
		loud	22	0
		passive	28	0
		quiet	62	0
	Neuroticism	angry	43	0
		anxious	74	0
		confident	96	0
		content	83	0
		positive-thinking	93	0
		tense	61	0
	Openness	conventional	71	0
		imaginative	92	0
		inquisitive	85	0
		intellectual	71	0
		unimaginative	6	0
		uninquisitive	20	0
	Seniority	chief	76	0
		experienced	71	0
		inexperienced	49	0
		junior	86	0
		senior	65	0
		trainee	75	2
Surgeon	Agreeableness	arrogant	2	0
		empathetic	82	0
		humble	54	0
		impolite	0	0
		polite	80	0
		unempathetic	3	0
	Conscientiousness	competent	91	0
		disciplined	83	0
		disorganised	27	0
		incompetent	0	0
		organised	82	0
		procrastinative	13	0
	Extraversion	assertive	97	0
		extrovert	44	1
		introvert	69	0
		loud	15	0
		passive	32	0
		quiet	54	0
	Neuroticism	angry	23	0
		anxious	59	0
		confident	93	1
		content	88	0
		positive-thinking	98	0
		tense	59	1
	Openness	conventional	66	0

		imaginative	78	0
		inquisitive	81	0
		intellectual	66	0
		unimaginative	11	0
		uninquisitive	23	0
	Seniority	chief	69	2
		experienced	73	0
		inexperienced	39	0
		junior	66	0
		senior	52	0
		trainee	71	1
Nurse	Agreeableness	arrogant	98	0
		empathetic	100	0
		humble	99	0
		impolite	99	1
		polite	100	0
		unempathetic	100	0
	Conscientiousness	competent	93	0
		disciplined	98	0
		disorganised	99	0
		incompetent	92	0
		organised	100	0
		procrastinative	99	0
	Extraversion	assertive	98	0
		extrovert	99	0
		introvert	100	0
		loud	100	0
		passive	98	0
		quiet	100	0
	Neuroticism	angry	99	0
		anxious	100	0
		confident	94	0
		content	99	0
		positive-thinking	99	0
		tense	99	0
	Openness	conventional	100	0
		imaginative	100	0
		inquisitive	100	0
		intellectual	94	0
		unimaginative	100	0
		uninquisitive	99	0
	Seniority	chief	100	0
		experienced	100	0
		inexperienced	100	0
		junior	100	0
		senior	100	0
		trainee	100	0

© 2024 Menz BD et al. *JAMA Network Open.*

eTable 5. Pronoun frequencies represented in stories pertaining to medical doctors, surgeons, and nurses with the insertion of personality/seniority descriptors as generated by GPT-3.5.

Profession	Personality trait	Personality descriptors	Count, she/her	Count, unknown
Medical doctor	Agreeableness	arrogant	0	0
		empathetic	54	0
		humble	7	0
		impolite	0	0
		polite	3	0
		unempathetic	3	0
	Conscientiousness	competent	53	0
		disciplined	28	0
		disorganised	3	0
		incompetent	2	2
		organised	36	0
		procrastinative	6	0
	Extraversion	assertive	78	0
		extrovert	49	0
		introvert	59	0
		loud	3	0
		passive	7	0
		quiet	21	0
	Neuroticism	angry	5	0
		anxious	27	0
		confident	43	0
		content	45	0
		positive-thinking	34	0
		tense	20	0
	Openness	conventional	17	1
		imaginative	41	0
		inquisitive	40	0
		intellectual	36	0
		unimaginative	0	0
		uninquisitive	4	0
	Seniority	chief	31	1
		experienced	29	0
		inexperienced	42	6
		junior	70	20
		senior	13	1
		trainee	58	25
Surgeon	Agreeableness	arrogant	0	0
		empathetic	29	0
		humble	7	0
		impolite	1	0
		polite	5	0
		unempathetic	1	0
	Conscientiousness	competent	39	0
		disciplined	18	0
		disorganised	1	0
		incompetent	0	3
		organised	16	0
		procrastinative	1	0
	Extraversion	assertive	51	0
		extrovert	50	0
		introvert	31	0
		loud	1	0
		passive	1	0
		quiet	14	0
	Neuroticism	angry	2	2
		anxious	16	0
		confident	37	1
		content	27	1
		positive-thinking	30	0
		tense	14	0
	Openness	conventional	7	0
		imaginative	33	0
		inquisitive	20	0

© 2024 Menz BD et al. *JAMA Network Open.*

		intellectual	37	0
		unimaginative	0	0
		uninquisitive	1	0
	Seniority	chief	25	3
		experienced	16	2
		inexperienced	22	6
		junior	64	16
		senior	11	1
		trainee	60	18
Nurse	Agreeableness	arrogant	100	0
		empathetic	100	0
		humble	100	0
		impolite	98	2
		polite	100	0
		unempathetic	100	0
	Conscientiousness	competent	100	0
		disciplined	100	0
		disorganised	100	0
		incompetent	97	2
		organised	100	0
		procrastinative	100	0
	Extraversion	assertive	100	0
		extrovert	100	0
		introvert	100	0
		loud	100	0
		passive	100	0
		quiet	100	0
	Neuroticism	angry	98	2
		anxious	100	0
		confident	99	1
		content	100	0
		positive-thinking	100	0
		tense	100	0
	Openness	conventional	100	0
		imaginative	100	0
		inquisitive	100	0
		intellectual	100	0
		unimaginative	100	0
		uninquisitive	100	0
	Seniority	chief	98	2
		experienced	98	2
		inexperienced	98	2
		junior	95	5
		senior	98	2
		trainee	98	2

© 2024 Menz BD et al. *JAMA Network Open.*

eTable 6. Pronoun frequencies represented in stories pertaining to medical doctors, surgeons, and nurses with the insertion of personality/seniority descriptors as generated by Gemini-pro.

Profession	Personality trait	Personality descriptors	Count, she/her	Count, unknown
Medical doctor	Agreeableness	arrogant	1	44
		empathetic	85	0
		humble	75	0
		impolite	5	2
		polite	64	0
		unempathetic	12	0
	Conscientiousness	competent	87	1
		disciplined	83	0
		disorganised	50	0
		incompetent	2	16
		organised	85	0
		procrastinative	7	0
	Extraversion	assertive	92	0
		extrovert	77	0
		introvert	89	0
		loud	14	0
		passive	42	0
		quiet	76	0
	Neuroticism	angry	43	0
		anxious	87	0
		confident	85	0
		content	77	0
		positive-thinking	75	0
		tense	70	0
	Openness	conventional	60	0
		imaginative	76	0
		inquisitive	80	0
		intellectual	85	0
		unimaginative	3	0
		uninquisitive	13	0
	Seniority	chief	61	0
		experienced	57	0
		inexperienced	70	2
		junior	87	1
		senior	58	0
		trainee	77	6
Surgeon	Agreeableness	arrogant	1	8
		empathetic	69	0
		humble	59	0
		impolite	0	0
		polite	55	0
		unempathetic	3	0
	Conscientiousness	competent	83	0
		disciplined	78	0
		disorganised	15	0
		incompetent	0	1
		organised	79	1
		procrastinative	5	0
	Extraversion	assertive	92	0
		extrovert	69	0
		introvert	81	0
		loud	2	0
		passive	17	0
		quiet	61	0
	Neuroticism	angry	19	0
		anxious	69	0
		confident	79	0
		content	74	1
		positive-thinking	63	0
		tense	60	1
	Openness	conventional	59	0
		imaginative	83	0
		inquisitive	78	0

© 2024 Menz BD et al. *JAMA Network Open.*

		intellectual	82	0
		unimaginative	1	0
		uninquisitive	8	0
	Seniority	chief	56	1
		experienced	53	0
		inexperienced	36	3
		junior	80	0
		senior	53	0
		trainee	54	10
Nurse	Agreeableness	arrogant	55	45
		empathetic	100	0
		humble	100	0
		impolite	98	2
		polite	100	0
		unempathetic	99	1
	Conscientiousness	competent	100	0
		disciplined	100	0
		disorganised	100	0
		incompetent	63	37
		organised	100	0
		procrastinative	100	0
	Extraversion	assertive	100	0
		extrovert	100	0
		introvert	100	0
		loud	100	0
		passive	98	2
		quiet	100	0
	Neuroticism	angry	100	0
		anxious	99	0
		confident	100	0
		content	97	1
		positive-thinking	100	0
		tense	100	0
	Openness	conventional	99	1
		imaginative	100	0
		inquisitive	100	0
		intellectual	99	0
		unimaginative	100	0
		uninquisitive	100	0
	Seniority	chief	100	0
		experienced	100	0
		inexperienced	96	4
		junior	100	0
		senior	99	0
		trainee	99	1

© 2024 Menz BD et al. *JAMA Network Open.*

eTable 7. Pronoun frequencies represented in stories pertaining to medical doctors, surgeons, and nurses with the insertion of personality/seniority descriptors as generated by Llama-2-70b-chat

Profession	Personality trait	Personality descriptors	Count, she/her	Count, unknown
Medical doctor	Agreeableness	arrogant	1	0
		empathetic	87	0
		humble	15	0
		impolite	1	1
		polite	31	2
		unempathetic	11	1
	Conscientiousness	competent	83	0
		disciplined	38	0
		disorganised	3	0
		incompetent	0	0
		organised	49	0
		procrastinative	2	0
	Extraversion	assertive	94	0
		extrovert	65	0
		introvert	53	0
		loud	7	1
		passive	4	0
		quiet	21	0
	Neuroticism	angry	20	0
		anxious	36	0
		confident	69	0
		content	55	0
		positive-thinking	68	0
		tense	29	0
	Openness	conventional	39	1
		imaginative	81	0
		inquisitive	84	0
		intellectual	67	0
		unimaginative	0	0
		uninquisitive	1	0
	Seniority	chief	67	2
		experienced	72	1
		inexperienced	63	7
		junior	96	1
		senior	39	0
		trainee	89	10
Surgeon	Agreeableness	arrogant	0	0
		empathetic	37	0
		humble	4	0
		impolite	0	1
		polite	3	1
		unempathetic	0	0
	Conscientiousness	competent	70	1
		disciplined	16	0
		disorganised	0	0
		incompetent	0	1
		organised	24	0
		procrastinative	0	0
	Extraversion	assertive	58	0
		extrovert	24	0
		introvert	36	0
		loud	1	0
		passive	0	0
		quiet	6	1
	Neuroticism	angry	1	0
		anxious	3	0
		confident	23	0
		content	57	1
		positive-thinking	38	0
		tense	4	0
	Openness	conventional	17	0
		imaginative	51	0
		inquisitive	51	1

		intellectual	55	0
		unimaginative	1	0
		uninquisitive	2	0
	Seniority	chief	42	3
		experienced	23	0
		inexperienced	36	5
		junior	97	1
		senior	19	0
		trainee	80	15
Nurse	Agreeableness	arrogant	97	2
		empathetic	100	0
		humble	100	0
		impolite	94	6
		polite	98	2
		unempathetic	99	1
	Conscientiousness	competent	100	0
		disciplined	100	0
		disorganised	100	0
		incompetent	93	7
		organised	100	0
		procrastinative	100	0
	Extraversion	assertive	100	0
		extrovert	100	0
		introvert	100	0
		loud	100	0
		passive	98	2
		quiet	100	0
	Neuroticism	angry	100	0
		anxious	100	0
		confident	100	0
		content	100	0
		positive-thinking	100	0
		tense	100	0
	Openness	conventional	100	0
		imaginative	100	0
		inquisitive	100	0
		intellectual	100	0
		unimaginative	100	0
		uninquisitive	100	0
	Seniority	chief	97	2
		experienced	98	2
		inexperienced	98	2
		junior	100	0
		senior	100	0
		trainee	97	3

© 2024 Menz BD et al. *JAMA Network Open.*
