# Supplementary material for: Gender Representation of Health Care Professionals in Large Language Model–Generated Stories
Source: JAMA Netw Open. 2024 Sep 23;7(9):e2434997. doi: 10.1001/jamanetworkopen.2024.34997 (PMC11420694; doi:10.1001/jamanetworkopen.2024.34997)
Supplement: Supplement 2. — Data Sharing Statement [file jamanetwopen-e2434997-s002.pdf]

Data Sharing Statement

Menz. Gender Representation of Health Care Professionals in Large Language Model–Generated Stories. *JAMA Netw Open*. Published September 23, 2024. doi:10.1001/jamanetworkopen.2024.34997

Data
Data available: Yes
Data types: Data (not involving human participants)
How to access data: Publicly accessible generative AI tools – including GPT-3.5-turbo, GPT-4, Gemini-pro, and Llama-2-70B-chat were used to generate the data evaluated in this manuscript. The data collated is provided in the Supplementary Appendix.
When available: With publication

Supporting Documents
Document types: None

Additional Information
Who can access the data: The research team would be willing to make the complete set of generated data (stories) available upon request from qualified researchers or policy makers whom submit a proposal detailing required access and intended use.
Types of analyses: The research team would be willing to make the complete set of generated data (stories) available upon request from qualified researchers or policy makers whom submit a proposal detailing required access and intended use.
Mechanisms of data availability: The research team would be willing to make the complete set of generated data (stories) available upon request from qualified researchers or policy makers whom submit a proposal detailing required access and intended use.
